# Dandy–Walker malformation and variants: clinical features and associated anomalies in 28 affected children—a single retrospective study and a review of the literature

**DOI:** 10.1007/s13760-022-02059-z

**Published:** 2022-09-06

**Authors:** A. Di Nora, G. Costanza, F. Pizzo, A. Di Mari, A. Sapuppo, A. Basile, A. Fiumara, P. Pavone

**Affiliations:** 1 Department of Clinical and Experimental Medicine, University of Catania Postgraduate Training Program in Pediatrics, Catania, Italy; 2 Department of Radiology, University of Catania Postgraduate Training Program in Radiology, Catania, Italy; 3 Department of Pediatric Neurology, University of Catania, Catania, Italy; 4 Radiology Unit, Policlinico G.Rodolico, Catania, Italy, University of Catania, 95123 Catania, Italy

**Keywords:** Neurology malformations, Epilepsy, Hydrocephalus

## Abstract

**Objective:**

To investigate the clinical characteristics, the neuroimaging features and associated anomalies observed in children affected by Dandy–Walker malformations (DWM) and variants (DWV) in a single tertiary hospital in Catania and compare our data to their existent in the literature.

**Methods:**

A retrospective case series using the medical records has been performed on 28 children diagnosed with DWM and DWV admitted to a single tertiary section of Pediatric Neurology, Department of Catania, Italy from January 2005 to January 2021. We reviewed the neuroimaging using the new diagnostic criteria of Klein et al.

**Results:**

Associated anomalies were frequently reported. Among these, hydrocephalus was found in 13/28 (48%), and hydrocephalus plus corpus callosum anomalies in three children (10%). We described corpus callosum, cardiac and genitourinary anomalies in 2/28 (7%), 3/28 (10%), and 3/28 (10%), respectively. The most common clinical features were the developmental delay and epilepsy observed in 19/28 (67%) and in 9/28 (32%) of the cases. The first exam at the diagnosis was MRI in 17/28 patients, followed by transfontanellar ultrasound in 5/28, computed tomography in 4/28 and prenatal ultrasound in 2/28. To note, a child with DWM was affected by Down syndrome and one by congenital disorders of N-linked glycosylation (CDG-IId).

**Conclusions:**

Children with DWV were more commonly observed than children with DWM. Hydrocephalus is an anomaly, frequently and equally reported in both DWM and DMV. Perinatal complications were frequent adverse events with severe respiratory distress and need for cardiopulmonary resuscitation. Cognitive involvement and epilepsy were the most common comorbidities. Single DWV is associated with a better developmental outcome.

## Introduction

**Fig. 1:**
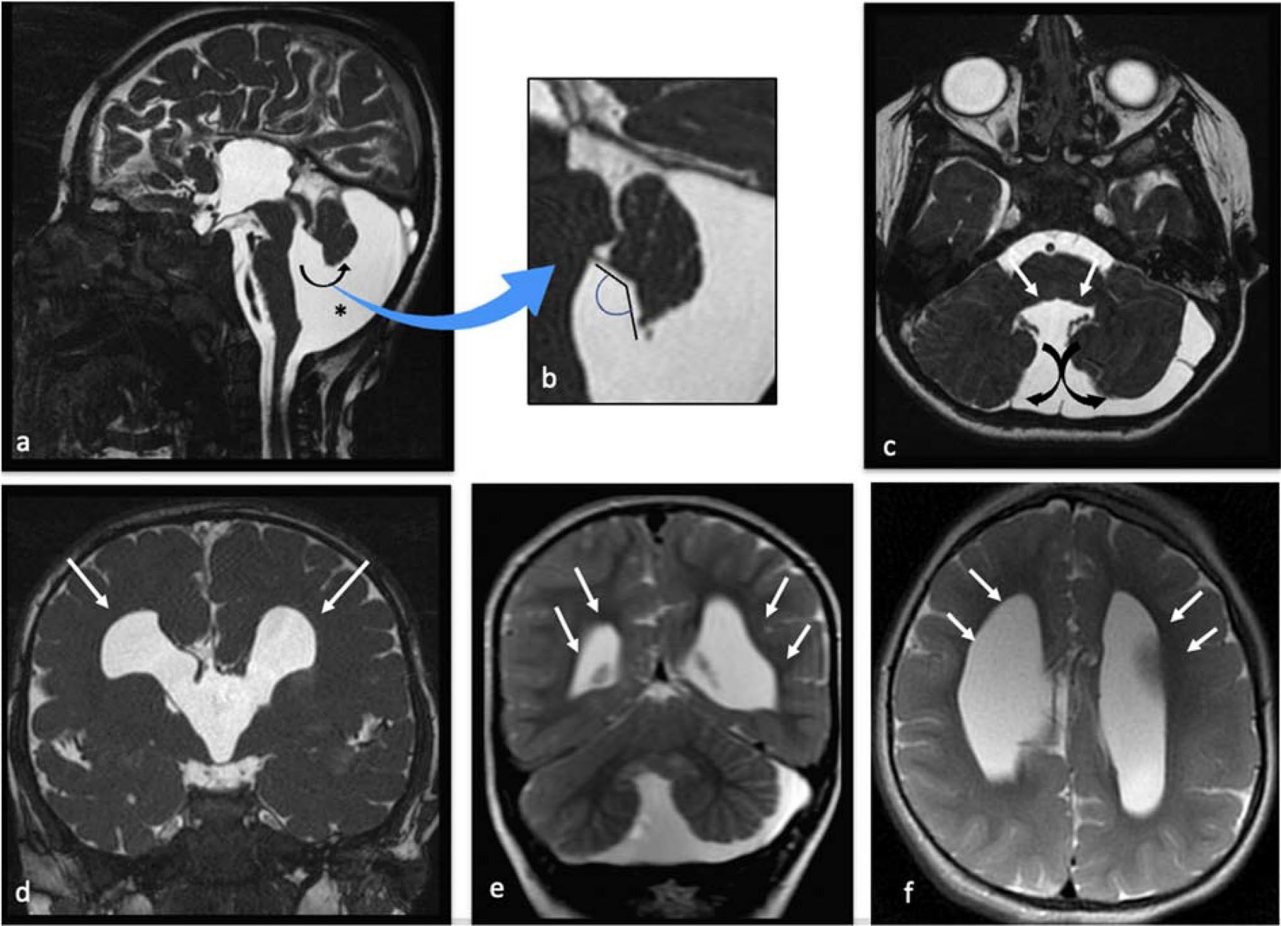
MRI of a 3-year-old boy with Dandy–Walker Variant (**a**–**c**) and associated corpus callosum agenesis with abnormal ventricular system (**d**–**f**). 3D FIESTA MR image **a** show posterior fossa cyst (star) widely communicating with the fourth ventricle, vermian hypoplasia (lower 1/4) with upward displacement of the vermis (curved arrow) and enlargement of the fastigium angle (**a**, **b**). Axial T2 weighted MR image shows anterolateral displacement of cerebellar hemispheres (curved arrow) and vermian hypoplasia (white arrow) (**c**). Coronal (**d**–**f**) and axial **f** T2W MRI shows corpus callosum agenesis with abnormal ventricular system (white arrows).

In 1887 there was the first description of DWM. Since then, new neuroimaging tools, such as MRI, allowed researchers to extend the knowledge on the anatomy of the posterior fossa and particularly of the cerebellar vermis. In 1989 Barkovich et al. proposed one of the most used radiological classification, defined the entity of “Dandy–Walker complex” and “Dandy–Walker spectrum” [1]. In 2003, Klein et al. reviewed the classification by Barkovich et al., focused the attention on these radiological criteria for the diagnosis [2] (Fig. 1).

1. Large, median posterior fossa cyst widely communicating with the fourth ventricle
2. Absence of the lower portion of the vermis at different degrees (lower 3/4, lower half, lower 1/4)
3. Hypoplasia, anterior rotation, and upward displacement of the remnant of the vermis
4. Absence or flattening of the angle of the fastigium
5. Large bossing posterior fossa with elevation of the torcular
6. Anterolateral displacement of normal or hypoplastic cerebellar hemispheres

It is important reserving the diagnosis of DWM to the cases that respond to the above-mentioned criteria by Klein et al., to avoid increasing confusion [3]. Differently to the DWM, DWV is characterized by mild vermian hypoplasia and a normal-sized posterior fossa with a small cystic cavity that communicates with the fourth ventricle [3–5]. The association of DWV with other congenital anomalies is largely limited to few reports.

The purpose of this retrospective case series was to analyze the clinical features and associated anomalies in children affected by DWM and DWV, reporting clinical differences between the two different groups.

## Methods

We reviewed the charts and MRI of 28 children between January 2005 and January 2021. The clinical aspects, the anomalies associated and the neurological outcome were also recorded.

Patients with DWM were identified by Klein’s criteria. (Fig. 1) Patients with DWV were identified by these characteristics: normal-sized posterior fossa, a posterior fossa cystic lesion that appeared to communicate with the fourth ventricle and mild inferior vermian hypoplasia. We report comorbidities associated with the anomalies, including other neurological anomalies, cardiac defects, genitourinary abnormalities. If possible, the presence of a chromosomal defect or syndrome association was documented.

## Results

We included in the study 28 patients, 4 with diagnosis of DWM and 24 with diagnosis of DWV. 11 patients were female and 17 were boys. The mean gestational age at the diagnosis was 12 months. No patients were premature, but 13 newborns (46%) at birth suffered by severe respiratory distress or other disturbances, which required treatment in neonatal intensive care. Nineteen patients (67%) were diagnosed based on findings of MR imaging studies, 5 based on ultrasonography (3 on transfontanellar US after birth, 2 in prenatal age), 4 on CT scans. No associated anomalies were reported in 10 children (35%). Hydrocephalus was described in 13 children (46%), carpus callosum anomalies in 2 (7%), hydrocephalus plus corpus callosum anomalies in 3 children (10%). Epileptic seizures were found in 9 (32%) children and developmental delay (DD) in 19 (67%). Cardiac abnormalities were seen in 3 (10%); cardiac defect consisted of patent ductus arteriosus, ventricular septal defect, and Fallot’s tetralogy. Gastrointestinal anomalies were observed in 1 child, who presented congenital diaphragmatic hernia associated with gastroesophageal reflux disease (GERD). Three children manifested genitourinary anomalies (cryptorchidism in 1 and pelvis ectasia in 2). In addition, 1 child was affected by panhypopituitarism, 1 by Down syndrome and 1 by CDG type II-d (Table 1)

**Table 1:** Description of associated abnormalities in (a) 24 patients with DWV, (b) 4 patients with DWM

Patient	Sex	Age at the diagnosis	Perinatal history	Radiological diagnostic exam	Other neuroradiological anomalies	Epilepsy	Outcome	Other sistemic defects
(a) 24 patients with DWV								
ID-01	M	7 years	Asphyxia at the birth, admission in NICU	MRI	Polymicrogiria, dysgenesia CC	Yes	DD	MRGE, cryptochidism
ID-02	F	3 months	unremarkable	CT scan	hydrocephalus	No	At the follow-up, DD	NR
ID-03	M	1 year	unremarkable	CT scan	hydrocephalus	No	DD	NR
ID-04	F	5 months	Admission in NICU for hypoglycemia in SGA	Transfontanellar US, confirmed by MRI	Hydrocephalus	No	DD	Pelvis ectasia, laryngomalacia
ID-05	M	1 year	Admission in NICU for feeding difficulties	Transfontanellar US, confirmed by MRI	no	No	No	No
ID-06	M	2 months	Admission in NICU for feeding difficulties	MRI	Hydrocephalus	No	DD	No
ID-07	M	2 years	Admission in NICU for distress and feeding difficulties	CT scan	Hydrocephalus	Yes	DD	
ID-08	F	3 months	Unremarkable	Transfontanellar US	No	No	DD in Trisomy 21	Trisomy 21, cardiac defect
ID-09	F	6 months	Admission in NICU for asphyxia	MRI	No	Yes	DD	No
ID-10	M	4 months	No	MRI	No	No	No	Pelvis ectasia
ID-11	M	Prenatal diagnosis	Admission in NICU for prenatal history	Suspected by prenatal US, confirmed by fetal MRI	Hydrocephalus	Yes	DD	Cardiac defect
ID-12	F	4 years	no	MRI	No	Yes	No	No
ID-13	M	Prenatal diagnosis	Admission in NICU for prenatal history	Suspected by prenatal US, confirmed by fetal MRI	Hydrocephalus	Yes	DD	No
ID-14	M	2 months	Admission in NICU for asphyxia	MRI	Hydrocephalus, agenesis CC	Yes	DD	Syndactily
ID-15	M	2 months	No	MRI	Hydrocephalus	No	DD	No
ID-16	F	5 months	No	MRI	Hydrocephalus, anomalies CC, cysts in inner ear	No	DD	No
ID-17	M	1 year	Admission in NICU for asphyxia	MRI	CC agenesis	Yes	No	No
ID-18	M	1.5 year	no	MRI	No	No	No	No
ID-19	M	15 years	no	MRI	Ventricular dilatation	No	DD	No
ID-20	M	2 years	no	MRI	No	No	DD	No
ID-21	M	3 years	No	MRI	No	No	No	No
ID-22	F	5 months	Admission in NICU for asphyxia	MRI	Anomalies CC, empty sella	No	No	Panipopituitarism
ID-23	F	8 months	Admission in NICU for asphyxia	MRI	No	No	DD	c.367G > A in CDG-Ig
ID-24	M	1 year	No	MRI	No	No	No	No
(b) 4 patients with DWV								
ID-25	F	4 months	Unremarkable	MRI	No	No	No	No
ID-26	M	1 year	Unremarkable	MRI	Hydrocephalus	yes	DD	No
ID27	M	1 year	Admission in NICU for asphyxia	MRI	Hydrocephalus	no	DD	Fallot’s tetralogy
ID28	F	12 years	Unremarkable	CT scan	No	No	DD	No

*CC* corpus callosum, *CDG-Ig* carbohydrate deficient glycoprotein syndrome type Ig, *CT* computed tomography, *DD* developmental delay, *MRI* resonance magnetic imagine, *NICU* unit intensive therapy, *US* ultrasound

## Discussion

DWM and DWV are rarely reported in literature [5–13]. The exact cause of these disorders remains unknown [5–8].

DWM and DWV are both cystic malformations of the posterior fossa. Cystic malformations are characterized by the presence of substantial cerebrospinal fluid (CSF) collection. Other cystic anomalies include persistent Blake’s pouch, mega cisterna magna, arachnoid cyst. The mega cisterna magna is usually a benign finding, while the other malformations could be associated with other congenital anomalies. DWM is associated with a large posterior fossa, while DWV is associated with a normal or small posterior fossa. The diagnosis is exclusively radiological. In Table 2 you can find the main characteristics of the posterior fossa malformations. In Tables 3 are synthetised the main characteristics and differences between DWM and DWV.

**Table 2:** Posterior fossa malformations

Cystic malformations	Noncystic malformations
Dandy walker malformations	Joubert syndrome
Dandy walker variant	Rhombencephalosynapsis
Persistent Blake’s pouch	Tectocerebellar dysraphia
Mega cisterna magna	Neocerebellar dysgenesis
Arachnoid cyst

**Table 3:** Main differences between the DWM and DWV

Dandy Walker malformations	Dandy Walker variant
Large, median posterior fossa cyst widely communicating with the fourth ventricle	Large, median posterior fossa cyst widely communicating with the fourth ventricle
Absence of the lower portion of the vermis at different degrees (lower ¾, lower half, lower ¼). Hypoplasia, anterior rotation, and upward displacement of the remnant of the vermis	Hypoplasia of the vermis, but complete in every portion
Large bossing posterior fossa with elevation of the torcular	Normal size of the posterior fossa

Many patients affected by DWM and DWV can remain clinically asymptomatic for years, while others may present with a variety of comorbidities leading to earlier diagnosis. Clinicians could investigate first symptoms related to the central nervous system, such as hydrocephalus, or could make diagnosis of other systemic symptoms such as cardiac defects, face, limbs and gastrointestinal or genitourinary system [5]. The disorder may have a clinically severe onset or to have a mild or almost asymptomatic course. In most cases the disturbances are present since birth or in the first year of life. Both the disorders the DWM and the DWV are often associated with extra and intracranial anomalies which have a relevant role in the outcome of the disorder. Children < 1 year of age often present with signs of hydrocephalus and increased intracranial pressure. Later, DWM and DWV usually presents with motor delay and incoordination. Spastic paraparesis is the most common motor deficit. The incidence of focal neurological deficits, such as nystagmus, cranial nerves palsies, truncal ataxia, explosive speech, and dysmetria, indicative of cerebellar or brain stem dysfunction, are relatively uncommon. Both DWM and DWV disorders are often associated with extra and intracranial anomalies. The incidence of focal neurological deficits, such as nystagmus, cranial nerves palsies, truncal ataxia, explosive speech, and dysmetria, indicative of cerebellar or brain stem dysfunction, are relatively uncommon. These symptoms may arise in case of persistence increased intracranial pressure. The cerebellar deficits, which usually affect axial movement rather than movements of the extremities tend markedly to improve after adequate control of hydrocephalus [1–3]. In older children with milder signs the disorders may present with macrocephaly, symptoms of intracranial hypertension, anomalous movements, ataxia, nystagmus, and episodes of headache. Cognitive disability and epileptic seizures may be observed. Several comorbidities may also correlate with DWM, including syndromic and non-syndromic, CNS and non-CNS anomalies, chromosomal abnormalities, cardiovascular conditions, mental illness, and severe intellectual disability [10].

**Fig. 2:**
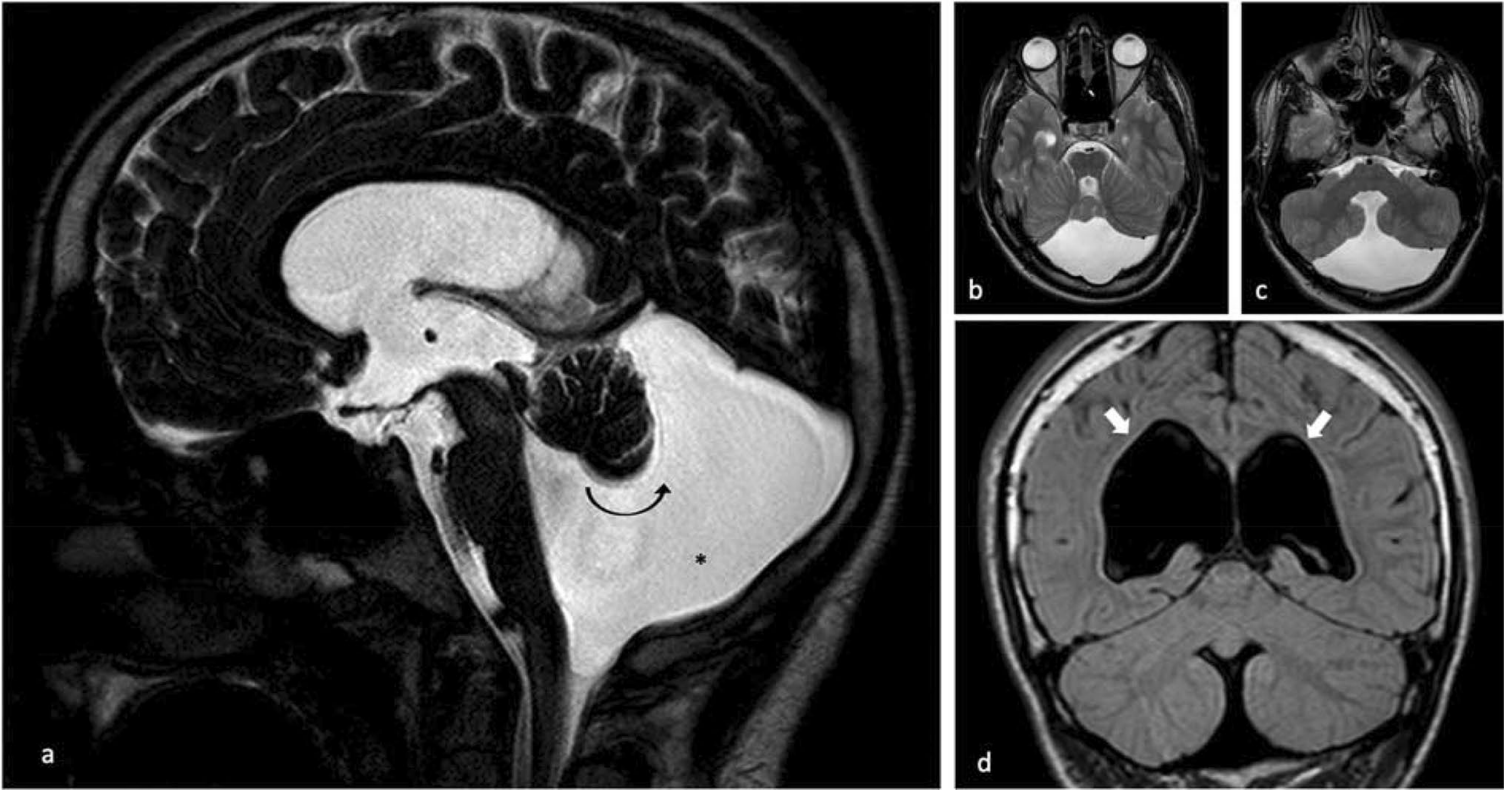
MRI of a 15-year-old boy with Dandy–Walker Variant. Sagittal 3D-DRIVE (**a**) Axial T2W (**b**, **c**) and coronal T1W (**d**) shows hypoplasia of the inferior vermis with anterosuperior rotation as seen on the sagittal scan (curved arrow), fourth ventricle dilatation and an enlarged posterior fossa (star). We can also see lateral ventricular dilatation (white arrows). There is no torcular-lambdoid inversion

**Fig. 3:**
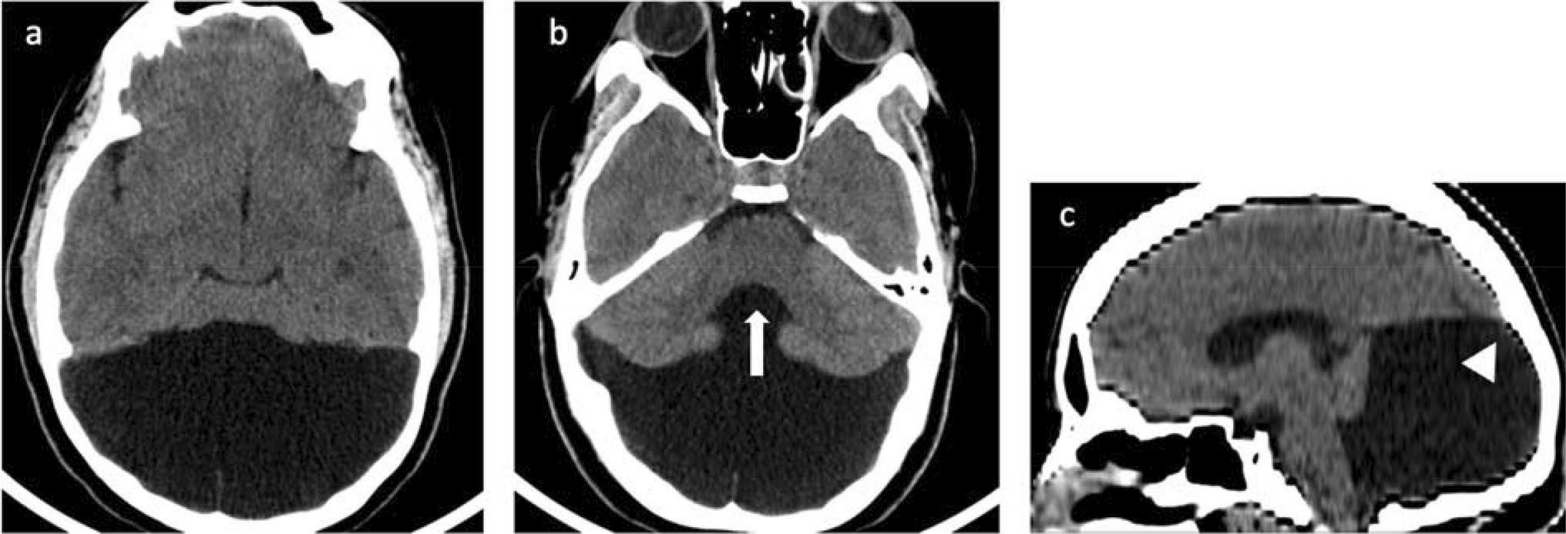
Axial non-enhanced computed tomography (NECT) scans (**a**, **b**) and sagittal MPR (**c**) in 12-year-old boy with Dandy–Walker malformation NECT shows all the main features of DWM: a large posterior fossa cyst, hypoplastic cerebellar hemispheres, and absence of the vermis (white arrow). Sagittal T1W image (**c**) show a massively dilated fourth ventricle, expanded posterior fossa, and elevated torcular herophili and transverse sinuses (arrowhead) and hypoplastic cerebellum

DWM and DWV are often associated with other brain malformations, such as hydrocephalus and anomalies of the corpus callosum, which have a relevant role in the outcome of the disorder. Our study reports that intracranial anomalies were present in the 54% of the population, confirming the data previous in literature. In literature, hydrocephalus has been observed in 50–80% rate of patients with DWM [5–10]. Differently, in DWV has not extensive reported. Our experience confirms that hydrocephalus is correlated with DWM and DWV, with the same frequency. In case of hydrocephalus, treatment is generally focused on alleviating hydrocephalus and posterior fossa symptoms, often including surgical interventions [8]. Treatment consists of treating the manifestations and associated comorbidities. Most patients present with signs and symptoms from increased intracranial pressure, most commonly related to hydrocephalus and posterior fossa cyst. For this reason, therapy generally aims to control intracranial pressure, usually through surgery [8]. Surgical treatment may include ventriculoperitoneal (VP), or cystoperitoneal (CP) shunts. Other patients may be candidates for endoscopic procedures, including endoscopic third ventriculostomy (ETV) [8]. In the current study, hydrocephaly occurred in 13/24 patients and approximately half required treatment with CSF diversion. Developmental delay was observed in 19 patients and of those 25% required significant assistance in their daily functioning. At the follow-up cognitive impairment and epileptic seizures were recorded with a frequency of 67% and 32%, respectively. In two of the children the cerebral malformation was part of two well defined disorders: one child presenting with the Down syndrome and the other with congenital disorder of glycosylation type II-d (CDG-IId). Although the structural cerebral anomalies of DWM and DWV are not strictly similar nevertheless share some common clinical features as high incidence in cognitive impairment and epilepsy and intracranial and extracranial associated anomalies. As a matter of facts, in this group of children we have found no notable differences in clinical features and outcome between the DWM and DWV and this in disagreement to what it has been reported by Sasaki et al. [5] who refer that development outcome is better in DWV than in DWM (Figs. 2, 3).

In contrast to the studies previous published, our casuistic reported DD in 67% of cases [7, 8, 13]. Other studies described this anomaly in only one third of the patients. In addition, Sasaki et al. reported that development outcome is better in DWV than in DWM [5]. Differently, our experience reports a negative outcome in both categories. Although, in according to the cited studies, our data confirm that contributing factors to poor neurological outcomes include the association with other CNS anomalies or neurological conditions.

Interestingly, we report that 13/28 patients were admitted in NICU for asphyxia or feeding difficulties. No other reported cases described this perinatal characteristic, probably due to a missing data in the materials’ study.

In addition, we report a patient affected by trisomy 21 and DWV. In literature, the coexistence of Down syndrome and DWM has been previously reported in few cases, suggesting that it is relatively uncommon [14, 15].

Finally, another rare association was a patient affected by carbohydrate deficient glycoprotein syndrome (CDG) and DWV. Peters et al. in 2002 reported a case of a 1.5 year old boy affected by CDG-I and DWM. No other cases were reported before our patient. So, it remains unclear if DWM/DWV are causally related to CDG or are a coincidental findings.

In the present study the frequency of isolated DWV has been more frequently observed than DWM. Clinical comparison among the clinical features presented by the disorders linked to DWM and DWV is not appropriate, considering the low frequency of patients with DWM in the present series. However, it has been noted that some features, such as hydrocephalus, cardiac anomaly, and cognitive impairment and epileptic seizures, were recorded in both the groups of children.

### Limits of the study

Our study had some limitations. The study population was small, especially for the DWM, but significant for a rare condition. All the data were obtained retrospectively. Our patients were only from a single tertiary hospital in Catania and genetic testing was not performed in all patients of our cohort.

## Conclusions

DWM and DWV are rare, complex, cerebral malformations both often associated with extra and intracranial anomalies. The study confirms that MRI is the election survey for the diagnosis of DWM and DWV. Although they are different radiological conditions, DWV and DWM share an high incidence in DD and intracranial and extracranial associated anomalies. The high frequency of comorbidities requires the coexistence of multidisciplinary specialists for early diagnosis and for rapid identification of the associated anomalies.

## Data Availability

The data used to support the findings of this study may be released upon application to the corresponding author, who can be contacted at alessandradinora@gmail.com.
